# New Mode of Taking Cod-Liver Oil

**Published:** 1853-01

**Authors:** 


*New mode of taking Cod-Liver Oil.* I have read Mr. Selwyn Morris's "New Mode of taking Cod-Liver Oil," and quite agree with his general principle of using a bitter infusion. I have been in the habit of recommending to my patients the use of pale or bitter ale as one of the best vehicles in which to take the oil, be it cod liver or castor. This description of ale being intensely bitter, and tonic to boot, from the large quantity of hops used in its manufacture, serves the purpose admirably; and another advantage is, that it can be obtained more readily than a quinine mixture or an infusion of quassia ; and moreover, being a stimulant, the stomach is also beneficially excited to retain and digest the fatty oil. As an extempore vehicle, I have frequently used the concentrated infusion of gentian (of course, diluted) with good effeet ; but when there is time to prepare an infusion, I would certainly give the preference to the quassia. *—*Canada Med. Jour.*

---

* Note.—We have received a letter recently from one of our patients who, whilst in Dublin, consulted Dr. Graves, was advised by him to take Cod-Liver Oil, in infusion of quassia, which, no doubt that eminent Physician has found to conceal the taste of that valuable though disagreeable remedy.

